# Comparison of 90-day complications and two-year reoperation rates between anterior and posterior interbody fusion for single-level degenerative spondylolisthesis

**DOI:** 10.1016/j.xnsj.2022.100127

**Published:** 2022-05-21

**Authors:** Stephen Georgiou, Satvir Saggi, Hao-Hua Wu, Lionel Metz

**Affiliations:** Department of Orthopedic Surgery, at the University of California at San Francisco, San Francisco, California

**Keywords:** Degenerative spondylolisthesis, Single-level, Anterior lumbar interbody fusion, Posterior lumbar interbody fusion, Reoperation, Complications, Readmission

## Abstract

**Background:**

For the surgical treatment of single-level degenerative spondylolisthesis (DS), patients can be treated with either an anterior or posterior interbody fusion. Prior studies have shown that patients with symptomatic degenerative spondylolisthesis treated surgically maintain substantially greater pain relief and improvement in function when compared to those treated non-operatively, but no consensus has emerged between which approach results in the best outcomes.

**Methods:**

The PearlDiver MARINER database was queried for patients with single-level DS who underwent either an anterior or posterior lumbar interbody fusion. Both populations were compared on multiple outcomes, including reoperation, post-operative complications, and readmission rates at 90 days, as well as rates of reoperation and cauda equina syndrome two-years postoperatively.

**Results:**

At 90 days patients who underwent anterior interbody were found to have higher rates of DVT (OR 2.53, 95% CI 1.74 – 3.70, p<0.001), ileus (OR 1.43, 95% CI 1.25 – 1.64, p<0.001), and readmission (OR 1.28, 95% CI 1.19 – 1.38, p<0.001). Patients who underwent posterior interbody fusion were found to have higher rates of revision procedures (OR 0.63, 95% CI 0.59 – 0.66, p<0.001), transfusion (OR 0.68, 95% CI 0.58 – 0.78, p<0.001), acute kidney injury (OR 0.84, 95% CI 0.75 – 0.95, p=0.0046), and cauda equina syndrome (OR 0.53, 95% CI 0.40 – 0.69, p<0.001). At 2 years, patients who underwent posterior fusion required revision procedures (OR 0.70, 95% CI 0.67 – 0.74, p<0.001) and developed cauda equina syndrome (OR 0.62, 95% CI 0.50 – 0.77, p<0.001) at a higher rate than those who underwent anterior fusion.

**Conclusions:**

Patients who underwent anterior interbody fusion for treatment of degenerative spondylolisthesis were found to have increased rates of DVT, ileus, and were more likely to be readmitted to the hospital within 90 days, while patients who underwent posterior interbody fusion were found to have higher rates of reoperation, transfusion, AKI, and cauda equina syndrome. Increased rates of reoperation and development of cauda equina in the posterior fusion group persisted at 2 years post-operatively.

## Introduction

Although degenerative spondylolisthesis (DS) is common in adults, with prevalence rates estimated at 2.7% in males and 8.1% in females[1], [ ]there remains no consensus regarding the best surgical approach for patients with DS. Prior studies have shown that surgical intervention can lead to improved outcomes for patients with DS [2,3], including the Spine Patient Outcomes Research Trial (SPORT) which found that patients with symptomatic degenerative spondylolisthesis treated surgically maintain substantially greater pain relief and improvement in function when compared to those treated non-operatively, with decompression and fusion appearing to be more effective than decompression alone.[4] Subsequent sub-analyses based off this trial found similar improvement based on surgical intervention for other populations. [5,6] However, no studies based on the SPORT trial looked at the effectiveness between anterior versus posterior interbody fusion for DS.

Interbody fusion techniques can be divided into anterior (anterior lumbar interbody fusion [ALIF], lateral lumbar interbody fusion [LLIF] and anterior to the psoas [ATP] interbody fusion) and posterior (posterior lumbar interbody fusion [PLIF] or transforaminal lumbar interbody fusion [TLIF]). The advantages of anterior interbody fusion include indirect decompression, allowing for maximal implant size/surface area facilitating aggressive correction of lordosis, and sparing of posterior spinal muscles, while posterior interbody fusion allows for direct decompression, improved visualization, and neural decompression without affecting anterior support structures.[7] While direct comparisons between the techniques have been completed [8], [9], [10], [11] complication rates between procedures vary by study and little is known about the long-term reoperation rates between anterior and posterior interbody fusions in the treatment of degenerative spondylolisthesis.

The aim of this study was to identify the key complications and readmission rates at 90 days post-operatively, as well as reoperation rates at two years post-operatively, between adult DS patients who underwent anterior or posterior interbody fusion.

## Methods

### Patient selection and study variables

Data for this study was collected from the PearlDiver All Payer Claims Database (MARINER), which is a for-fee database. Included in the database are claim records from 2010-Q2 2018 across all payer types for over 121 million distinct patients. Claim data includes ICD-9 & ICD-10 diagnosis coding, ICD-9 & ICD-10 procedural coding, CPT procedural coding, prescription NDC coding, demographic, physician specialty, and geographic region or state. Access to the database was provided by PearlDiver Technologies and was stored on a remote server managed by PearlDiver. Our study was IRB exempt as a retrospective database study using de-identified patient data from a HIPAA-compliant healthcare database, causing no more than minimal risk to subjects involved.

Patient records were collected from a subset of the PearlDiver database that included all patients with a claim for spinal fusion. Those with degenerative spondylolisthesis were filtered for based on ICD-9 code 7384. Two study groups were then created from this population of patients. The anterior interbody fusion group, which included patients who underwent ALIF, LLIF, or ATP, was selected for with ICD-9 code 8106 and CPT code 22558. Within this group, those with any instance of posterior fusion (ICD-9-8107, 8108, CPT-22630) during the same hospitalization were excluded. The posterior interbody fusion group, which included patients who underwent PLIF or TLIF, was selected for with ICD-9 code 8108 and CPT code 22630. Within this group, those with any instance of anterior fusion (ICD-9-8106, CPT-22558) for the same hospitalization were excluded. From these groups, patients were also excluded if they had a code for multilevel fusion, non-lumbar fusion, fusion for fracture treatment, malignant neoplasm, congenital muscle deformities, or multi-level laminectomy from the same day as the interbody fusion [Appendix I]. Groups were tested to ensure they are mutually exclusive.

Both populations were queried for age, sex, and pre-existing co-morbidities including acute myocardial infarction, asthma, chronic obstructive pulmonary disease (COPD), cancer, cerebrovascular disease, coronary artery disease (CAD), diabetes mellitus (DM), hypertension, obesity, osteoarthritis, renal disease, and tobacco use. Data on 90-day reoperation, complication, and readmission rates were collected. Reoperation procedures included device removal, refusion, debridement, incision and drainage, exploration of prior fusion, removal of instrumentation, and spinal decompression procedures (full list of reoperation codes in Table 1). We also collected data on the breakdown of reoperation codes that each patient qualified for both at 90 days as well as at 2 years. Complications compared include deep vein thrombosis (DVT), ileus, transfusion, acute kidney injury (AKI), postoperative hematoma, wound complications, cauda equina syndrome, and major medical complications (cardiac arrest, pneumonia, pulmonary embolism). At two years we continued to trend reoperation procedures and rates of cauda equina syndrome but excluded other complications that were less likely to have occurred due to the primary operation.

**Table 1:** Reoperation Codes

**ICD9**	
P-0309	Other exploration and decompression of spinal canal
P-7869	Removal of implanted devices from bone, other bones
P-8130	Refusion of spine, not otherwise specified
P-8134	Refusion of dorsal and dorsolumbar spine, anterior column, anterior technique
P-8135	Refusion of dorsal and dorsolumbar spine, posterior column, posterior technique
P-8136	Refusion of lumbar and lumbosacral spine, anterior column, anterior technique
P-8137	Refusion of lumbar and lumbosacral spine, posterior column, posterior technique
P-8138	Refusion of lumbar and lumbosacral spine, anterior column, posterior technique
P-8139	Refusion of spine, not elsewhere classified
P-8622	Excisional debridement of wound, infection, or burn
**CPT**	
22015	Incision and drainage, open, of deep abscess (subfascial), posterior spine; lumbar, sacral, lumbosacral
22830	Exploration of spinal fusion
22850	Removal of posterior non-segmental instrumentation
22852	Removal of posterior segmental instrumentation

### Statistical analysis

Pearson's χ2 analysis was used to compare the anterior interbody fusion and posterior interbody fusion surgical groups in regard to demographics and comorbidities. Logistic regression analysis was subsequently used to perform multivariate analyses in terms of assessing differences in 90-day and 2-year complications between the anterior and posterior groups while also adjusting for CCI score, sex, and age. The Bonferroni correction was utilized and the threshold of statistical significance was set at α=0.00556. All statistical analysis was performed with R (The R Project for Statistical Computing) through the PearlDiver Database software.

## Results

Overall, the anterior interbody fusion group consisted of 14,971 (62.40% female) patients while the posterior interbody fusion group consisted of 36,648 patients (64.79% female) (Table 2). The percentage of patients who were at or above the age of 75 at the time of surgery was significantly higher in the anterior versus posterior group (8.21% vs 6.86%). With regard to comorbidities, patients in the anterior interbody fusion group were found to have significantly greater rates of asthma. In contrast, patients in the posterior interbody fusion group were found to have significantly greater rates of prior myocardial infarction, chronic obstructive pulmonary disease, cancer, cerebrovascular disease, coronary artery disease, diabetes, hypertension, osteoarthritis, and renal disease. No statistically significant difference in rates of obesity or tobacco usage between the two groups was found.

**Table 2:** Patient Characteristics/Comorbidities of Anterior Interbody Fusion and Posterior Interbody Fusion Groups

	Anterior fusion patients		Posterior fusion patients		
	14,971		36,648		
Demographics	Number of patients	%	Number of patients	%	p-value
*Male*	5,629	37.60%	12,904	35.21%	**<0.001**
*Female*	9,342	62.40%	23,744	64.79%	
**Age ≥ 75 yr**	1,229	8.21%	2,514	6.86%	**<0.001**
**Comorbidities**					
*Asthma*	2,583	17.25%	5,820	15.88%	**<0.001**
*COPD*	5,849	39.07%	15,075	41.13%	**<0.001**
*Cancer*	2,370	15.83%	6,625	18.08%	**<0.001**
*Cerebrovascular* *Disease*	3,718	24.83%	10,233	27.92%	**<0.001**
*Coronary Artery* *Disease*	4,463	29.81%	12,431	33.92%	**<0.001**
*Diabetes*	6,284	41.97%	16,831	45.93%	**<0.001**
*Hypertension*	11,594	77.44%	30,136	82.23%	**<0.001**
*Obesity*	6,642	44.37%	16,345	44.60%	0.634
*Osteoarthritis*	8,722	58.26%	23,035	62.85%	**<0.001**
*Prior MI*	1,541	10.29%	4,466	12.19%	**<0.001**
*Renal Disease*	2,714	18.13%	7,793	21.26%	**<0.001**
*Tobacco Use*	7,043	47.04%	17,233	47.02%	0.97

The number of reoperation procedures at 90 days and 2 years based on individual ICD9/CPT codes were collected and are shown in Table 3.

**Table 3:** Breakdown of Patients Who Underwent Reoperation Based on Code

	90 Day		2 Year	
ICD-9	Anterior	Posterior	Anterior	Posterior
P-0309	575	3,600	828	4,651
P-7869	405	1,353	766	2,472
P-8130	0	0	0	0
P-8134	0	0	0	0
P-8135	20	21	46	52
P-8136	85	28	168	103
P-8137	233	331	454	786
P-8138	70	190	111	340
P-8139	0	0	0	0
P-8622	30	108	51	161
**CPT**				
22015	88	205	118	235
22830	299	591	595	1055
22850	150	369	283	656
22852	188	457	374	794
**Revisions**	1,547	5,706	2,536	8,101
**All patients**	14,971	36,648	14,971	36,648

Table 3 shows the number of patients who had each code in their medical record in the 90 day or 2 year period following primary procedure. Patients could have multiple codes in their records.

### 90-day reoperation, complication, and readmission rates

**Fig. 1:**
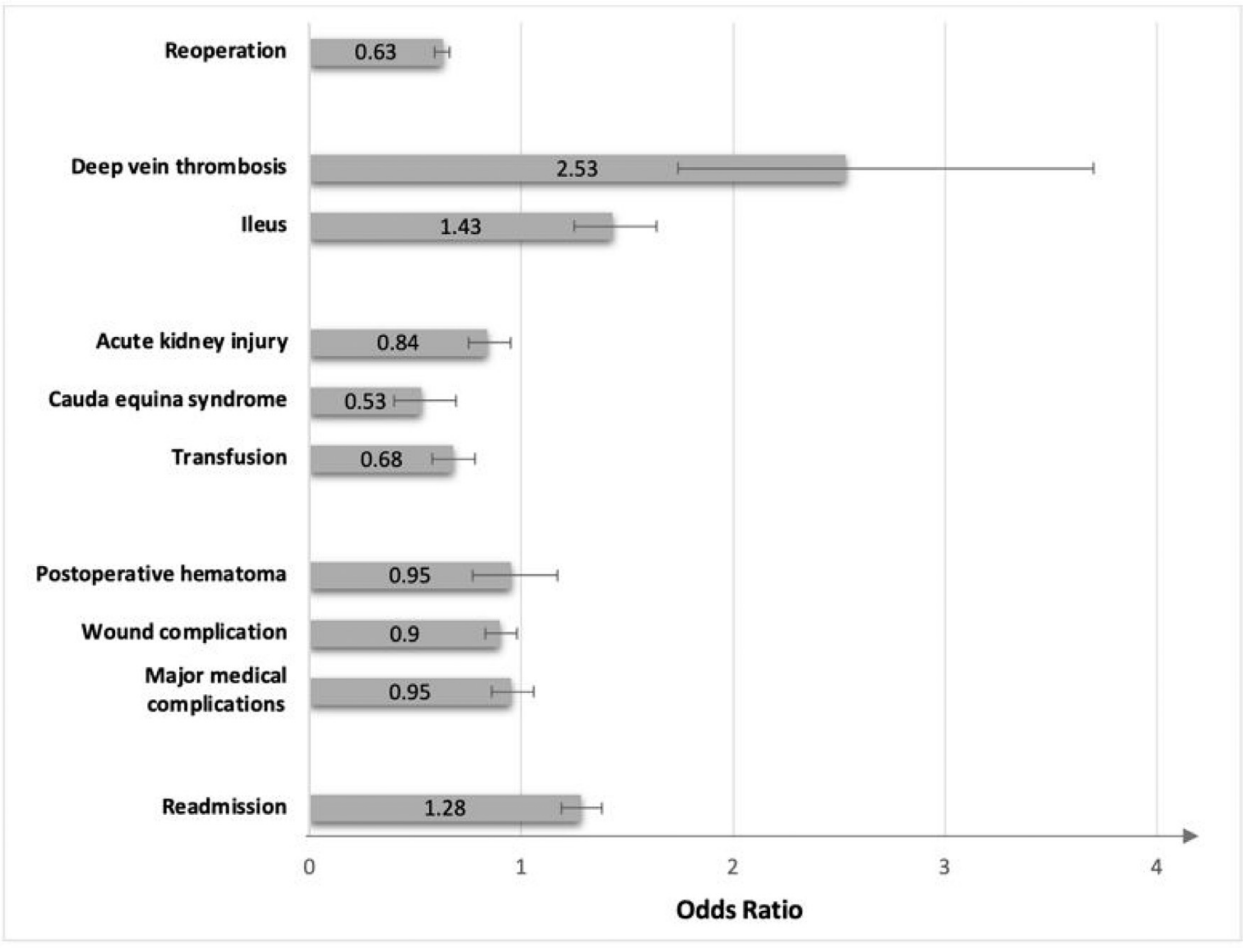
Adjusted Odds Ratios for Reoperations and Complications at 90 Days Post-Operatively Between Anterior and Posterior Interbody Fusion Groups

After adjusting for age, CCI, and gender, patients in the anterior fusion group were found to have lower rates of reoperation at 90 days than those in the posterior fusion group (OR 0.63, 95% CI 0.59 – 0.66, p<0.001) (Figure 1, Table 4). Patients in the anterior interbody fusion group were found to have higher rates of deep vein thrombosis (OR 2.53, 95% CI 1.74 – 3.70, p<0.001) and ileus (OR 1.43, 95% CI 1.25 – 1.64, p<0.001) in the 90-day postoperative period compared with patients in the posterior interbody fusion group. However, rates of transfusion (OR 0.68, 95% CI 0.58 – 0.78, p<0.001), acute kidney injury (OR 0.84, 95% CI 0.75 – 0.95, p=0.0046), and cauda equina syndrome (OR 0.53, 95% CI 0.40 – 0.69, p<0.001) were higher in the posterior interbody fusion group. There was no significant difference in the likelihood of a wound complication, postoperative hematoma, or major medical complications between the anterior interbody fusion group and the posterior interbody fusion group at 90 days postoperatively.

**Table 4:** Adjusted Odds of Reoperation Procedures, Complications, and Readmissions at 90 Days Post-operatively in Anterior Compared with Posterior Interbody Fusion

	Anterior	Posterior	OR	95% CI	p-value
Reoperation	1,547 (10.3%)	5,706 (15.6%)	0.63	0.59 – 0.66	<0.001
Deep vein thrombosis	65 (0.4%)	51 (0.1%)	2.53	1.74 – 3.70	<0.001
Ileus	350 (2.3%)	589 (1.6%)	1.43	1.25 – 1.64	<0.001
Acute kidney injury	421 (2.8%)	1,077 (2.9%)	0.84	0.75 – 0.95	0.0046
Cauda equina syndrome	67 (0.5%)	276 (0.8%)	0.53	0.40 – 0.69	<0.001
Transfusion	257 (1.7%)	944 (2.6%)	0.68	0.58 – 0.78	<0.001
Postoperative hematoma	125 (0.8%)	301 (0.8%)	0.95	0.77 – 1.17	0.64
Wound complication	793 (5.3%)	1,918 (5.2%)	0.90	0.83 – 0.98	0.01
Major medical complications	588 (3.9%)	1,376 (3.8%)	0.95	0.86 – 1.06	0.37
Readmission	1,345 (9.0%)	2,447 (6.7%)	1.28	1.19 – 1.38	<0.001

Total	14,971	36,648			

A significantly greater proportion of patients in the anterior group were readmitted within 90 days compared to the proportion of patients in the posterior group (OR 1.28, 95% CI 1.19 – 1.38, p<0.001).

### Two-year reoperation and cauda equina syndrome rates

**Fig. 2:**
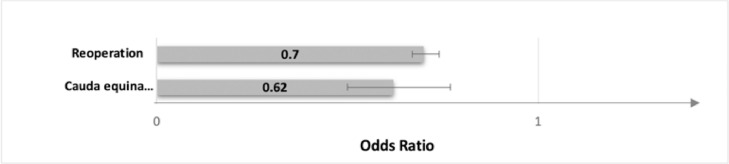
Adjusted Odds Ratios for Reoperations and Cauda Equina Syndrome at 2 Years Post-Operatively Between Anterior and Posterior Interbody Fusion Groups

At two years postoperatively, patients who underwent anterior fusion were found to have undergone reoperation procedures at a lower rate than those who had posterior fusion, with 16.9% of patients in the anterior fusion group undergoing reoperation procedures and 22.1% of patients in the posterior fusion group (OR 0.70, 95% CI 0.67 – 0.74, p<0.001) (Figure 2, Table 5). In addition, patients who had posterior lumbar fusion were more likely to have developed cauda equina syndrome (OR 0.62, 95% CI 0.50 – 0.77, p<0.001) in the two years after the primary procedure.

**Table 5:** Adjusted Odds of Reoperation and Cauda Equina Syndrome at 2 Years Post-operatively in Anterior Compared with Posterior Interbody Fusion

	Anterior	Posterior	OR	95% CI	p-value
Reoperation	2536 (16.9%)	8101 (22.1%)	0.70	0.67 – 0.74	<0.001
Cauda equina syndrome	108 (0.7%)	373 (1.0%)	0.62	0.50 – 0.77	<0.001

Total	14,971	36,648			

## Discussion

In this paper we review the differences in reoperation, readmission, and complication rates in patients with single-level DS who underwent anterior or posterior interbody fusion. Both anterior and posterior approaches for interbody fusion are regularly employed in clinical practice, each with their own benefits and risks. While there have been studies performed looking at complications between lumbar fusion approaches, no strong consensus regarding which approach is better for these patients has emerged amongst the orthopedic community, with current differences in treatment choices likely stemming from limited available scientific evidence, different specialty and training backgrounds, and different practice cultures based on geographic region and practice setting.[12]

This study aims to provide data on complication rates for both approaches in patients with degenerative spondylolisthesis using one of the largest healthcare databases in the world. To our knowledge this is the largest study done comparing anterior and posterior interbody fusion for patients with single-level degenerative spondylolisthesis and the largest that tracks patients with DS undergoing interbody fusion two years post-operatively.

The data from our study suggests that more patients with symptomatic DS were treated with posterior interbody fusion than anterior interbody fusion from 2010 to 2018. Anterior interbody fusion, while newer and possessing its own intrinsic benefits, remains less prevalent than posterior fusion for the treatment of DS. We found that patients who underwent anterior interbody fusion had a higher rate of DVT, ileus, and readmission at 90 days post-operatively.

The increase in DVT development in patients undergoing fusion from an anterior approach could possibly be due to manipulation of vasculature intraoperatively, while higher rates of post-operative ileus are likely explained by the approach through the abdominal musculature in anterior interbody fusion. Other studies looking at lumbar interbody fusions had similar findings. Manunga et al looked at complications for patients undergoing ALIF and had 1.7% of patients in the study develop venous thromboembolism and 3.1% develop post-operative ileus.[13] Another paper by Shillingford et al. found that anterior approaches have a higher risk of perioperative DVT in patients with degenerative lumbar disc disease or spondylolisthesis.[14] Qureshi et al reported that at 30 days readmission rates were much higher in patients who underwent ALIF than in those who underwent PLIF/TLIF.[15] The higher readmission rates didn't correlate with a higher reoperation rate, so while revision surgeries weren't statistically different between the two groups, those undergoing anterior interbody fusion returned to the hospital at a higher rate.

For patients who underwent posterior interbody fusion, we found higher rates of revision procedures, transfusion, acute kidney injury, and cauda equina syndrome. Several other studies found similar results. Fleege et al showed that patients with L5/S1 isthmic spondylolisthesis who underwent PLIF had a higher rate of revisions of fusion and wound revisions.[8] A paper by Liu et al. looking at perioperative complications between TLIF and PLIF found that PLIFs were associated with statistically significant higher rates of revision procedures, as well as had increased incidence of nerve root injury and blood transfusion, but did not compare PLIFs with anterior fusions.[16] A literature review by Guigui et al. discussing various surgical treatment options for DS notes that PLIF have longer operative times, increased blood loss, and increased risk of neurological complications[1], while a meta-analysis by Teng et al found that PLIF had the greatest blood loss when compared to other lumbar interbody fusions.[17] Shillingford et al. showed that patients with degenerative disc disease or spondylolisthesis had a significantly greater need for blood transfusions 72 hours after PLIF/TLIF in a univariate analysis, although the results did not remain significant in a multivariate analysis.[14] A comparative study by Pradhan et al. found that patients who underwent anterior fusion had significantly less blood loss, operative time, and need for transfusion.[18]

The information gathered in this study may be useful in guiding surgeons’ decision for anterior versus posterior fusion technique depending on patient-specific pre-operative risk factors and which intra-operative risks will be best tolerated. However, there are some limitations to this study. There does not exist a unique code for LLIF and anterior to the psoas interbody fusion to distinguish from ALIF, so this entire category of interbody fusion is grouped under “anterior.” The posterior interbody fusion group also grouped together PLIF and TLIF. In addition, due to the selection of the patient populations in the search query, any patients who underwent 360-degree index operation cases were not analyzed. This type of study also does not allow us to deduce whether indication for anterior vs posterior approach depends on surgeon preference or patient-specific factors. This is a retrospective database study, and codification errors, failure to report or overreporting of adverse events, and biases in patient selection could influence the results. As such the data cannot be used to conclude causation and future prospective trials are needed for further inquiry on these two approaches to interbody fusion.

However, the results reported in our study are based on an exceptionally large number of spine patients with real-world data from different centers with varying levels of surgical expertise, strengthening the application of our data to clinical practice. Our data also corroborated with several other smaller studies comparing interbody fusion techniques in various spinal pathologies, reinforcing the validity of the results.

## Conclusion

Surgeons can perform lumbar interbody fusions from either an anterior or posterior approach. The anterior approach was found in our study to be associated with increased rates of readmission, DVT, and ileus with decreased rates of revision procedures, transfusion, AKI, and cauda equina syndrome at 90 days post-operatively when compared with posterior interbody fusion. In addition, at 2 years there was a lower rate of reoperations and cauda equina in the anterior fusion group. The results from this study provide more information on some of the short-term and long-term outcomes for each approach, and this information may be useful in guiding surgeons’ decisions on which approach to employ based on patient-specific risk factors. However, future prospective research individually comparing the types of anterior and posterior approaches is warranted.

## Funding

No funding was obtained for this study.

## Role in the Study

Study concept and design (HHW, LM); acquisition of data (SG); analysis and interpretation of data (SG, SS, HHW); drafting of the manuscript (SG, SS, HHW); critical revision of the manuscript for important intellectual content (SG, SS, HHW, LM); statistical analysis (SG, SS); administrative, technical, or material support; study supervision (SG, SS, HHW, LM).

## Disclosure

The authors of this manuscript have no conflicts of interest to disclose.

## Funding disclosure

No funding was obtained for this study.

## Conflicts of Interest

There are no conflicts of interest to disclose.

## Appendix Supplementary materials

Image, application 1
