# Supplementary material for: Comparison of 90-day complications and two-year reoperation rates between anterior and posterior interbody fusion for single-level degenerative spondylolisthesis
Source: N Am Spine Soc J. 2022 May 21;10:100127. doi: 10.1016/j.xnsj.2022.100127 (PMC9144004; doi:10.1016/j.xnsj.2022.100127)
Supplement: Supplementary file 1 [file mmc1.docx]

**Appendix I – Exclusion Criteria**

| **ICD9** |  |
| --- | --- |
| D-1922 | Malignant neoplasm of spinal cord |
| D-1923 | Malignant neoplasm of spinal meninges |
| D-1983 | Secondary malignant neoplasm of brain and spinal cord |
| D-2253 | Benign neoplasm of spinal cord |
| D-2254 | Benign neoplasm of spinal meninges |
| D-2375 | Neoplasm of uncertain behavior of brain, unspecified |
| D-7542 | Congenital musculoskeletal deformities of the spine |
| D-8163 | Fusion or refusion of 4-8 vertebrae |
| D-8164 | Fusion or refusion of 9 or more vertebrae |
| **CPT** |  |
| 22600 | Arthrodesis, posterior or posterolateral technique, single level, cervical below C2 |
| 22610 | Arthrodesis, posterior or posterolateral technique, single level, thoracic |
| 22802 | Arthrodesis, posterior, for spinal deformity, with or without cast, 7 to 12 vertebral segments |
| 22804 | Arthrodesis, posterior, for spinal deformity, with or without cast |
| 22595 | Arthrodesis, posterior technique, atlas-axis (C1-C2) |
| 22590 | Arthrodesis, posterior technique, craniocervical (occiput-C2) |
| 22556 | Arthrodesis, anterior interbody technique, including minimal discectomy to prepare interspace (other than for decompression), thoracic |
| 22585 | Arthrodesis, anterior interbody technique, including minimal discectomy to prepare interspace (other than for decompression), each additional interspace |
| 22554 | Arthrodesis, anterior interbody technique, including minimal discectomy to prepare interspace (other than for decompression), cervical below C2 |
| 22325 | Open treatment and/or reduction of vertebral fracture(s) and/or dislocation(s), posterior approach, 1 fractured vertebra or dislocated segment, lumbar |
| 22326 | Open treatment and/or reduction of vertebral fracture(s) and/or dislocation(s), posterior approach, 1 fractured vertebra or dislocated segment, cervical |
| 22327 | Open treatment and/or reduction of vertebral fracture(s) and/or dislocation(s), posterior approach, 1 fractured vertebra or dislocated segment, thoracic |
| 22328 | Open treatment and/or reduction of vertebral fracture(s) and/or dislocation(s), posterior approach, 1 fractured vertebra or dislocated segment, each additional level |
| 63017 | Laminectomy with exploration and/or decompression of spinal cord and/or cauda equina, without facetectomy, foraminotomy or discectomy (eg, spinal stenosis), more than 2 vertebral segments |
